# External debt and economic growth in Sub-Saharan Africa: Does governance matter?

**DOI:** 10.1371/journal.pone.0264082

**Published:** 2022-03-04

**Authors:** Charles O. Manasseh, Felicia C. Abada, Ebelechukwu L. Okiche, Ogochukwu Okanya, Ifeoma C. Nwakoby, Peter Offu, Anuli R. Ogbuagu, Chiedozie O. Okafor, Paul C. Obidike, Nnenna G. Nwonye

**Affiliations:** 1 Department of Banking & Finance, University of Nigeria, Enugu Campus, Nsukka, Nigeria; 2 Social Science Unit, School of General Studies, University of Nigeria, Nsukka, Nigeria; 3 Department of Jurisprudence & Legal Theory, University of Nigeria, Enugu Campus, Nsukka, Nigeria; 4 Department of Banking and Finance, Institute of Management and Technology, Enugu, Nsukka, Nigeria; 5 Department of Political Science, Alex Ekwueme Federal University, Ndufu-Alike, Kwo, Abakaliki, Nigeria; 6 Department of Economic & Development Studies, Alex Ekwueme Federal University, Abakaliki, Nigeria; 7 Department of Psychology, Alex Ekwueme Federal University, Ndufu-Alike, Kwo, Abakaliki, Nigeria; 8 Department of Accounting/Banking & Finance, Alex Ekwueme Federal University, Ndufu-Alike, Abakaliki, Nigeria; Szechenyi Istvan University: Szechenyi Istvan Egyetem, HUNGARY

## Abstract

This study empirically examined the impact of external debt on economic growth. Also, the interactions of governance, external debt and external debt volatility were further investigated with emphasize on the interective effect of governance as proxied by Kaufmann, D., (2007) quality governance measures such as; government effectiveness, political stability, voice and accountability, regulatory quality and corruption control on economic growth. The study utilized annual time series data, focusing on thirty selected Sub-Saharan African (SSA) countries for the period 1997 to 2020. The Dynamic System Generalised Method of Moments estimation technique was adopted while controlling for conventional sources of economic growth. Empirical findings from the study reveal that external debt and external debt volatility have a negative and significant impact on economic growth in SSA. Furthermore, the interaction of governance indicators, external debt and its volatility, had a positive impact on economic growth in SSA. This study recommends that SSA government should endeavor to avoid excessive external debt to promote the regions’ capacity to invest in her financial prospects, and to circumvent the danger of repayment of loans using her small income. The SSA governments should also improve the quality of governance by ensuring political stability, minimising corruption, implementing sound policies and regulations that can permit and promote economic growth through the development of the private sector. The governments must ensure that every borrowed debt is properly supervised and utilised for its purposes to spur economic growth. More so, the Guidotti-Greenspan rule of Reserve adequacy should be applied to keep excess borrowings in check.

## 1. Introduction

External debt has been an important source of finance for most developing countries, mainly as a way of supplementing local revenue sources for development purposes. These countries mostly have low levels of domestic savings which have made borrowing a necessity [1]. Argued that when tax revenues are limited and government is reluctant to compromise macroeconomic stability by printing more money, borrowing is an attractive option for financing infrastructural development projects. According to [2], public borrowing occurs both in domestic and foreign markets however, its misuse in the domestic market can lead to financial instability and the crowding out of the private sector. As a result, countries utilise mostly foreign borrowings for capital projects. However, if these debts are not used for the right productive activities, countries might end up worse-off financially with dire consequences for both immediate and long-run macroeconomic conditions. For this reason [3], argues that excessive debt has constituted an obstacle to sustainable economic growth and poverty reduction, and as such subjects many developing countries particularly Sub–Saharan Africa to severe economic crisis resulting from excruciating debt burden. While debt has always been perceived a universal phenomenon [4], it has more adverse effects on developing economies like Sub–Saharan Africa, than the western economies [5]. Similarly [6], pointed that while external debt promote economic growth, it may however discourage investments through its crowding-out effect, due to charaterise increasing rates of interest that may likely results to a significant reduction in total private investment spending. This reduction in private investiment dampens the initial increase in total investment spending. Also, evidence has shown that the severity of debt burden is high in public debt owed to non-residents repayable in foreign currencies, than private debt [7]. In as much as [8] further reiterated the effect of public debt burden in most developing countries [9], classified the rationale for external debt and its effect into external and internal factors. External factors include the cumulative effects of world price shocks which create fiscal imbalances that require huge borrowings to manage, worsening terms of trade and liberal lending policies of international banks. Internal factors are attributed to excessive monetary expansion which causes inflation, over-reliance on external borrowing, over-valued exchange rate and poor management of public projects.

The debate on the impact of external debt on economic growth has been empirically investigated with conflicting findings. On the one hand, when external debt is properly utilised, it promotes productivity and income such that the ensuing benefits help to offset the accrued debt, resulting in a positive net effect on the recipient economy. Conversely, external debt may retard economic growth if the funds are not channelled towards the right productive activities. The economy may be affected through overcrowding, debt overhang, and liquidity constraints, amongst others [10]. Asserts that external debt retards investment while [11–14], argued that the impediment on investment results from slow economic growth due to external debt shocks. According to [14], using available funds for debt servicing rather than for domestic investment purposes may subject an economy to growth risk. Though [15], revealed that a statistically significant relationship existed between debt service and growth in Sub–Saharan Africa, and findings from [16] were contradictory. In 2009, Africa had an external debt of around US$ 300 billion, with the region spending about 16 percent of the continent’s export earnings on debt servicing. A look at South Africa and Nigeria, two of the strongest economies in Sub–Saharan Africa, shows that the external debt stock in the year 2000 was approximately 20 percent and 80.45 percent of their Gross National Income (GNI), respectively [7]. By the year 2005, external debt stock had reduced to 17.69 percent for South Africa, but increased again to 29.5 percent in the year 2010, and further increased to 45.21 percent in the year 2015 [7]. In contrast, Nigeria’s external debt stock gradually declined to 26.04 percent in the year 2005, and further decreased in the year 2010 to just 4.43 percent of GNI. By 2015, Nigeria’s external debt stock stood at 6.23 percent [7].

As previously stated, an increasing debt burden retards investment spending and by extension, economic growth. The decrease in the rate of economic growth further reduces the rate of investment which could lead to a continuous downward spiral in an economy. Hence, considering the fact that external debt is rarely investigated with deep analysis in Sub-Saharan Africa, this study therefore examined the impact of external debt on economic growth in thirty selected Sub-Saharan African countries. Furthermore, we extended the inquiry to examine the effect of external debt volatility on economic growth, and also ascertain the interactive effect of governance and external debt on economic growth of the selected countries. This study departs from previous studies in several ways. First [5, 17–20], studied the relationship between external/public debt and economic growth. Their findings show a significant relationship existing between debt and growth. However, these studies do not control for the influence of governance or institutional environment on external debt considering the fact that it’s a major problem in Sub-Saharan Africa. This is important because evidence has shown that countries with transparent government or quality institutions are less corrupt (see [21]), and such countries benefit more from utilising external debt compared to countries with weak institutional quality [22]. Second, other studies such as [23, 24] investigated the nexus between public debt, corruption and economic growth. While [23] found a positive and significant impact of corruption on external debt-growth [24], argued from the perspective of shadow economy, which was found to be positively related to corruption in public debt. However, these studies do not take into consideration factors that may likely curb corruption such as regulatory quality, and government effectiveness, etc. Third, studies by [25, 26] are most closely related to ours. While [25] focused on MENA countries [26]; studied external debts, institutions and growth in SSA. In [26], the measure of institutional quality or governance proposed by [27] was adopted, just like in our study. However, unlike [26], we further examined the influence of external debt volatility to account for exogenous shocks. Additionally, since quality governance or institution is perceived to be an important indicator that can strongly influence macroeconomic environment as well as economic growth [28], it is pertinent to interact each of the five measures of governance with external debt and evaluate their impact on economic growth of the selected countries. Hence [26], ignored the interative influence of institution and external debt on growth, but accounted for the impact of institution on economic growth. In this study, external debt volatility is used to account for the influence of external debt shocks on economic growth. In line with [22, 28], emphasises the importance of viable institutions or effective government in explaining the divergence in economic performances across developing economies. The remaining part of the paper includes a discussion on the external debt profile in Sub–Saharan Africa and quality of governance in sections two and three respectively, while section four provides a brief review of related literature. The methodology for the study is discussed in section five. Results from the analysis are presented and discussed in section six, while we conclude with relevant policy recommendations in section seven.

## 2. External debt profile in Sub-Saharan Africa

The debt burden facing most African countries has been a great hindrance to their growth and development, and deepened their poverty level with the consequent low living standards. In 2012, 33 Sub–Saharan African countries were classified as heavily indebted poor countries [29]. According to the International Labour Organisation (ILO), the huge burden of Sub-Saharan Africa’s debt is a serious obstacle to employment creation and growth as investment resources, which should be used for productive pursuits, are diverted to meet external debt service obligations. Table 1 below presents the external debt profile in Sub–Saharan Africa for the years 2008 to 2020. The table shows that total external debt stock which was US$3,576,358.8 million in 2008 jumped to US$3,849,726.9 million in 2009, and further increased to US$4,533,538.0 million in 2010. By 2011, SSAs total external debt stock amounted to US$5,298,841.2 million. Note that between 2012 and 2014, the debt stock increased by US$1207079.4 million as a result of continuous external borrowing. External debt stock decreased to US$6,604,487.7 million in 2015 but then increased to US$6,876,978.0 million in 2016, which further decreased to US$577,634.10 million in 2017. But since the recorded fall in debt stock experience in 2017, there has been consistent increase in external debt stock in Nigeria. As such, external debt stock grew from US$613,463.0 million recorded in 2018 to US$664,966.8 and US$701,945.1 million in 2019 and 2020 respectively. According to Central Bank of Nigeria, the increase in external debt stock is motivated by astronomical increase in external debt. Factors such as increasing public expenditure growth, especially expenditure on capital projects, loan from the international community at non-concessional interest rates, over reliance on imports, which results to emergence of trade arrears are considered major causes of debt situation in Nigeria, while rising movements in the interest rate influenced the extent of external debt stock.

**Table 1:** External debt profile, Sub-Saharan Africa, 2008–2020.

Year	External debt Stock US$ (million)	Long-term external debt US$(million)	Use of IMF credit US$(million)	External debt stocks to export (%)	External debt stocks to GNI (%)	Debt service to exports (%)	Short-term debt to external debt stocks (%)
2008	3,576,358.8	2,740,536.1	49,251.3	63.3	21.4	9.4	22.0
2009	3,849,726.9	2,884,963.5	145,044.3	85.2	23.5	11.6	21.3
2010	4,533,538.0	3,130,581.6	154,407.7	80.5	22.6	9.5	27.5
2011	5,298,841.2	3,574,599.5	154,426.8	76.4	22.4	8.3	29.6
2012	5,872,326.0	4,004,957.3	146,106.5	81.0	23.3	8.6	29.3
2013	6,643,120.3	4,443,213.8	128,392.9	89.0	24.9	9.3	31.2
2014	7,079,405.4	4,755,020.8	113,902.4	93.1	25.5	10.4	31.2
2015	6,604,487.7	4,767,014.0	113,693.2	97.9	25.0	12.0	26.1
2016	6,876,978.0	5,052,221.4	115,199.9	106.7	26.0	14.2	24.9
2017	577,634.10	489,615.10	20,584.90	149.7	37.1	11.9	11.7
2018	613,463.0	519,349.00	21,616.50	142.4	37.7	15.5	11.8
2019	664,966.8	567,039.70	22,946.70	155.6	39.6	16.1	11.3
2020	701,945.1	588,901.20	41,357.50	205.1	43.7	21.2	10.2

Source: World Bank International Debt Statistics (2021).

Long-term external debt which is a subset of total external debt stock stood at US$2,740,536.1 million in 2008 and increased to US$3,130,581 million in 2010. It further increased to US$4,767,014 million and US$5,052,221 million in 2015 and 2016 respectively [29]. Though, the increasing long-term external debt recorded in 2015 and 2016 decreased to US$489,615.10 million in 2017 but further increased to US$519,349.00 million in 2018. Furthermore, in 2019, it rose from US$567,039.70 million to US$588,901.20 million in 2020 [29]. The use of IMF credit amounted to US$154,407.7 million in 2010 and increased by US$19.1 million from 2010 to 2011. IMF credit decreased to US$146,106.5 million in 2012 and later fell to US$113,902.4 million in 2014 before rising to US$115,199.9 million in 2016. Thus, in 2017, the use of IMF credit drastically reduced to US$20,584.90 million, which slightly increased from US$21,616.50 million in 2018 to US$22,946.70 million in 2019. Hence, in 2020, it increased to US$41,357.50 million, which is almost double 2019 record.

**Fig 1:**
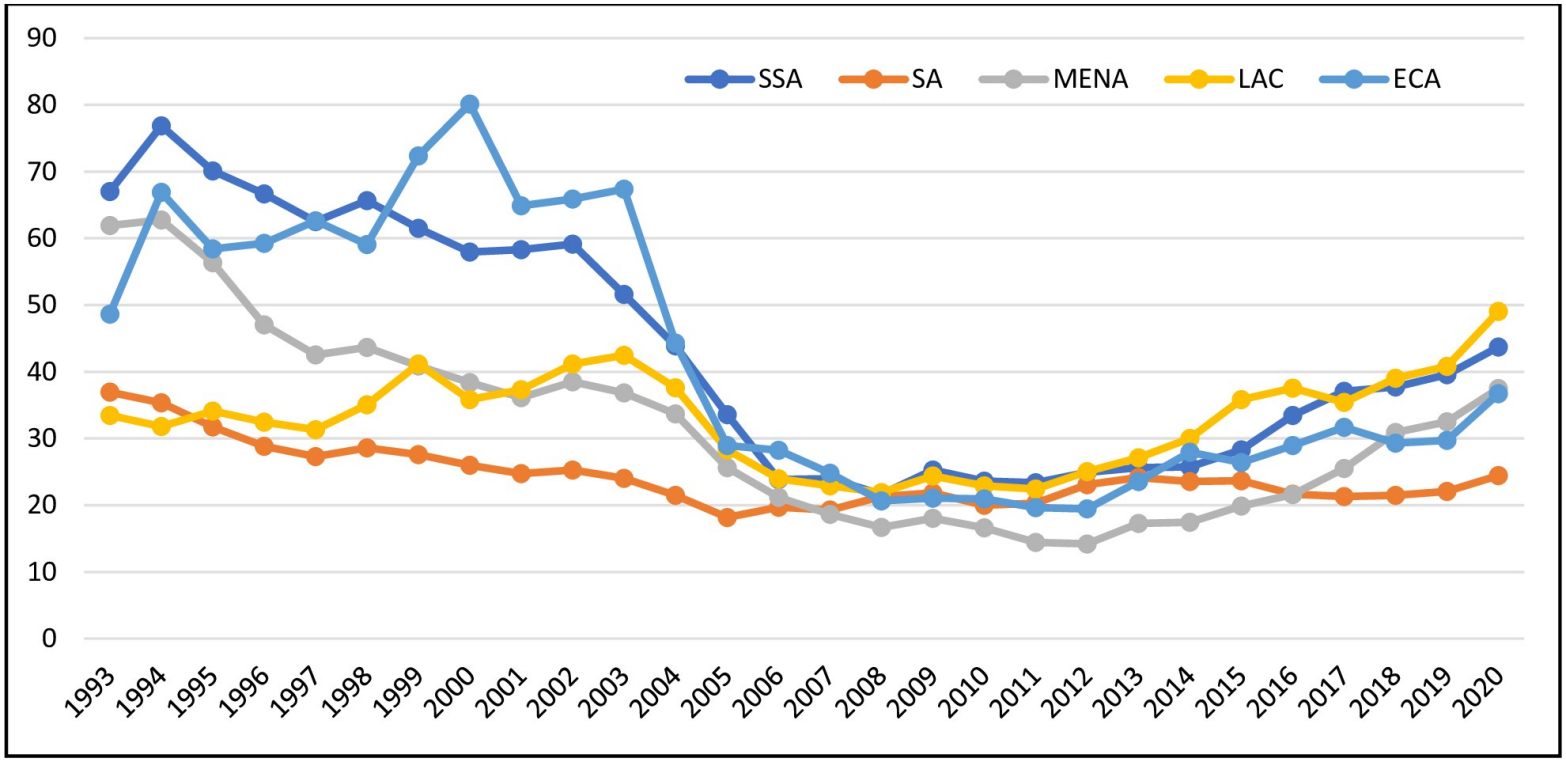
External debt (% of GNI) across regions (1993–2020). Source: WDI (2020). Note: SSA denotes Sub-Saharan Africa; SA denotes South Asia; MENA denotes Middle East and North America; LAC denotes Latin America and the Caribbean; ECA denotes Europe and Central Asia. Regional data excludes high income countries except for South Asia.

External debt stocks to exports stood at 85.2 percent in 2009 and decreased to 76.4 percent in 2011. They further increased to 81 percent in 2012 and 89 percent in 2013 [19]. External debt stocks to exports had been on the increase over the years (2012–2020). In 2016, it stood at 106.7 percent, and increased from 149.7 percent recorded in 2017 to 205.1 percent in 2020. Similarly, external debt as a percent of GNI owed to official creditors for the pre-crisis period (2004–2008) averaged 17.6 percent. This fell to 22.6 percent and 22.4 per cent in 2010 and 2011 respectively, before increasing to 23.3 percent in 2012. It stood at 24.9 percent in 2013, and was estimated at 25.5 percent in 2014 and 25.0 percent in 2015 [7]. Hence, since 2015, external debt as a percent of GNI slightly increased to 26.0% in 2016, but surged to 37.1% in 2017 through 39.6% and 43.7% recorded in 2019 and 2020 respectively. The debt service to export, measured by the ratio of actual debt service payments to exports of goods and services, averaged 12.15 percent between the years 2008 to 2020, while short-term debt as a percentage of external debt stock averaged 22.16 percent between the years 2008 to 2020. Fig 1 gives a clear picture of external debt in Sub-Saharan Africa in comparison to the rest of the world. The figure shows that external debt in Sub-Saharan Africa was the highest in the world between the years 1993 to 2004 which supports the conclusion of the study by [30, 31] that external debt had a negative effect on economic growth.

External debt in Europe and Central Asia became the highest in the world from the year 2005 to 2015. Between these years, external debt averaged 26.06 percent in Sub-Saharan Africa, amounting to 21.18 percent in South Asia, 16.39 percent in the Middle East and North Africa, 26.30 percent in Latin America and the Caribbean, and 41.90 percent in Europe and Central Asia [32].

### 3. Quality governance and external debt in Sub-Saharan Africa

At a fundamental level, governance determines how society, organisations or institutions manage their affairs and resource endowments. Hence, according to Merriam Webster, governance is the “act of governing or overseeing the control and direction of a country or an organization”. Also, it can perceived as the procedure for decision making and its implementation. According to [33], governance is also: “the traditions and institutions by which authority in a country is exercised. It includes the process by which governments are selected, monitored and replaced”, as well as “the capacity of the government to effectively formulate and implement sound policies and; the respect of the citizens, state for the institutions that govern economic and social interactions among them.” Based on this definition [33], identified six measures of quality governance which include: (a) corruption control, (b) regulatory quality, (c) government effectiveness, (d) voice and accountability, (e) political stability and absence of violence and, (f) rule of law, which form part of the Worldwide Governance Indicators (WGI).

Effective policy implementation in any country is strongly dependent on the viability of the legal system, typically reflected in the extent of law enforcement and order in the land [22]. Since quality governance has become a major tool for sustainable growth and development purposes around the world, the establishment of a favourable macroeconomic environment requires policies that incorporate Kaufmann’s governance indicators. Quality governance ensures impartiality, corruption control, government effectiveness in implementation of policies, voice and accountability in all government activities, and improved regulatory quality through the consistent enforcement of rules. All these are necessary conditions for the type of macroeconomic environment that can foster increase in savings, investment and economic growth [28]. However, in many economies in Africa, the quality of governance has degenerated since gaining their independence, and this has become a disturbing state of affairs. The increasing external debt burden and looming debt crisis in the region has become a source of anxiety for many analysts. A report by the African Economic Outlook [34] revealed that the 60% debt-to-GDP threshold established by the African Monetary Co-operation Program (AMCP) was exceeded by a total of 6 African countries from 2010 to 2017 and, about 15 countries in 2018 and 2019, 23 countries in 2020, 22 countries as at October 2021, and 22 countries forecasted in 2022 (see Table 2). Likewise, the 55% debt-to-GDP ratio proposed by the International Monetary Fund (IMF) was equally exceeded by 8 countries from 2010 to 2017, 18 countries in 2018, 21 countries in 2019, 23 countries in 2020, 25 countries in 2021 and forecasted 23 countries in 2022 (see Table 2). According to [35], these countries may find it difficult to service future debt repayments, with grave implications, for their borrowing costs and government bond yields.

**Table 2:** Sub-Saharan Africa government external debt (% of GDP), 2021.

SSA Countries	Government Debt (% of GDP)					
	2010–17	2018	2019	2020	2021	2022
Angola	46.1	93.0	113.6	136.5	103.7	90.8
Benin	26.2	41.1	41.2	46.1	52.3	48.9
Botswana	18.5	15.7	16.3	19.5	22.8	27.2
Burkina Faso	28.5	38.0	42.0	46.5	48.2	48.9
Burundi	42.5	53.0	60.3	67.0	72.4	71.2
Cape Verde	105.3	125.6	124.9	158.1	160.7	152.1
Cameroon	23.7	39.6	42.3	45.8	45.8	43.8
Central African Republic	43.6	50.0	47.2	44.1	46.5	44.0
Chad	38.1	49.1	52.3	47.9	44.0	44.3
Comoros	19.2	16.9	19.5	22.3	26.6	29.9
Congo, Democratic Republic of the	21.1	15.1	15.0	15.2	11.9	10.1
Congo, Republic of	55.5	77.1	81.7	101.0	85.4	76.9
Côte d’Ivoire	33.3	36.0	38.8	47.7	50.2	51.1
Equatorial Guinea	18.8	41.2	43.0	48.9	42.7	45.4
Eritrea	173.5	185.6	189.3	184.9	175.1	159.3
Eswatini	17.8	33.9	40.0	41.2	46.0	50.9
Ethiopia^1^	48.7	61.1	57.9	55.4	57.1	
Gabon	37.6	60.9	59.8	77.4	72.1	63.7
The Gambia	63.5	83.6	83.0	83.5	82.3	79.1
Ghana	45.1	62.0	62.6	78.9	83.5	84.9
Guinea	44.1	39.3	38.4	43.8	47.5	45.8
Guinea-Bissau	53.1	59.2	65.9	79.3	79.1	78.1
Kenya	42.6	57.3	59.0	67.6	69.7	70.2
Lesotho	39.8	49.6	50.6	50.4	50.0	50.2
Liberia	25.3	40.1	54.8	61.9	56.6	54.8
Madagascar	36.4	40.4	38.5	46.0	48.8	49.3
Malawi	31.4	43.9	45.3	54.7	59.3	65.4
Mali	28.8	36.1	40.6	47.4	51.0	50.6
Mauritius	59.8	66.2	84.6	96.9	101.0	99.8
Mozambique	66.6	107.1	105.4	128.5	133.6	127.6
Namibia	31.0	50.4	59.6	65.3	69.9	72.6
Niger	23.6	36.9	39.8	45.0	48.6	49.5
Nigeria	18.7	27.7	29.2	35.0	35.7	36.9
Rwanda	27.7	44.9	50.2	60.1	74.8	78.2
São Tomé & Príncipe	80.8	93.9	71.6	81.4	60.7	59.1
Senegal	41.0	61.5	63.8	68.7	71.9	70.1
Seychelles	73.0	59.1	57.7	96.5	81.9	82.8
Sierra Leone	45.8	69.1	71.7	73.7	71.1	68.0
South Africa	41.0	51.6	56.3	69.4	68.8	72.3
South Sudan	41.2	46.3	31.3	35.8	64.4	35.1
Tanzania	34.3	40.5	39.0	39.1	39.7	39.6
Togo	45.5	57.0	52.4	60.3	62.9	62.6
Uganda	24.5	34.8	37.0	44.1	49.1	50.2
Zambia	40.2	80.4	97.4	128.7	101.0	106.8
Zimbabwe^2^	44.9	61.5	113.9	86.1	54.0	60.3
Sub-Saharan Africa	33.5	47.5	50.4	57.3	56.6	56.4
Oil-exporting countries	25.4	41.9	43.9	48.8	46.1	44.5
Oil-importing countries	39.3	50.7	54.1	61.8	62.2	63.2
Middle-income countries	32.7	47.7	50.9	59.6	58.6	58.8
Low-income countries	37.0	46.8	49.0	50.4	50.4	49.0
Countries in fragile situations	36.3	41.7	46.8	49.7	46.9	46.3

Sources: IMF African Regional Economic Outlook, October 2021.

The devastating debt crisis had left many countries in Africa vulnerable to macroeconomic shocks due to misappropriation of the accumulated debt. According to Debt Overhang Theory by [6], “if the probability that the future debt will be greater than the repayment ability of a country, the costs of expected debt-service could inhibit further domestic and foreign investment”. This appears to be the situation in Africa where some countries borrowed externally without proper cost-benefit analyses. Though, many analysts have argued that poor governance and improper conducts in the process of ensuring compliance with laws, regulations, rules, standards, and social norms is the cause of the rising debt servicing and increasing debt burden in Africa. Hence, Governments attempt to effectuate successful implementation of policies by enforcing laws and regulations adequately will promote efficient allocation of the external borrowing and economic growth.

## 4. Debt and the myth of the debt-trap narrative in Africa

**Fig 2:**
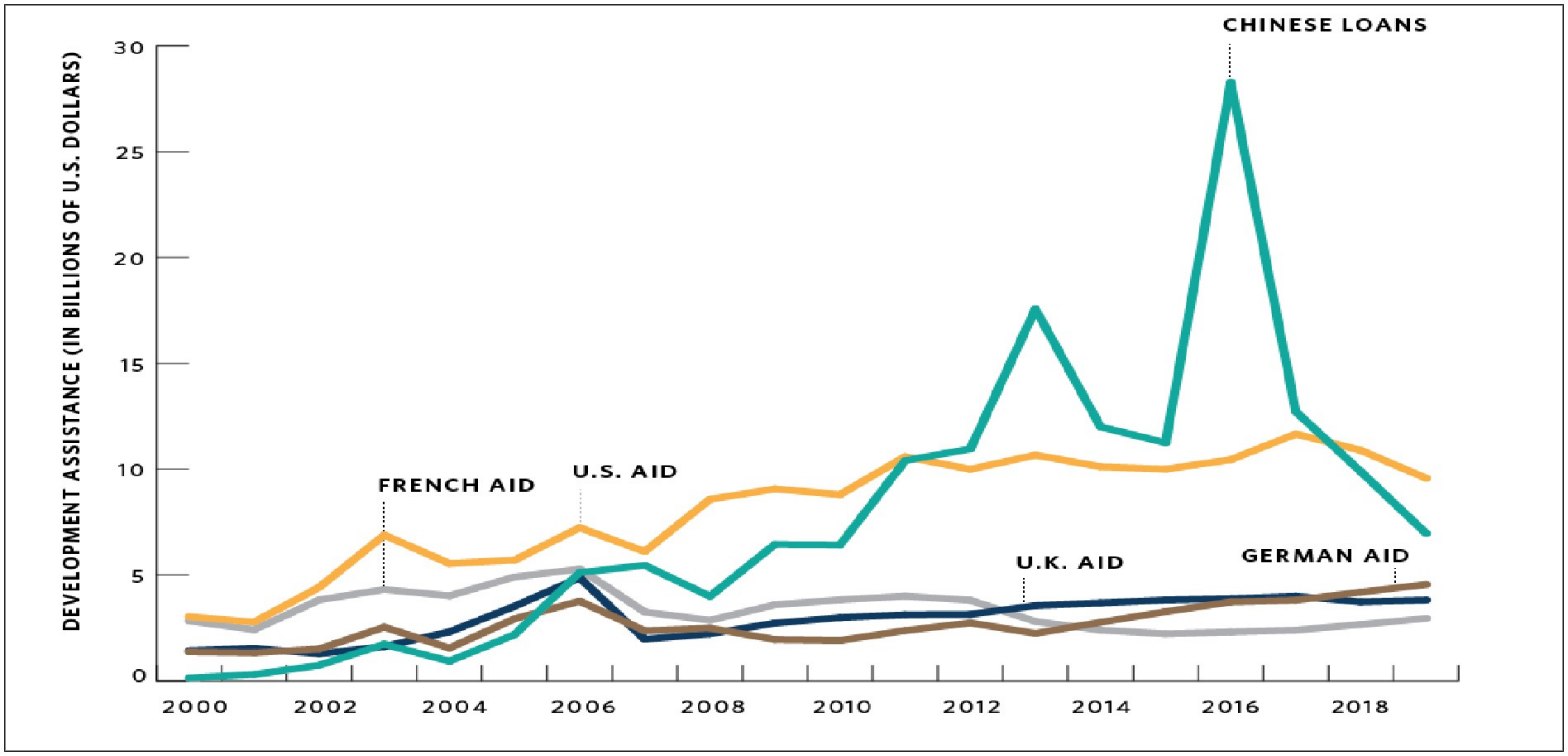
Plots of China’s lending and other aid in Africa. Source: Chinese Loans to Africa Database version 2.0 (2021), retrieved from https://chinaafricaloandata.bu.edu/; Total Official Development Flows by Country and region (ODF) and OECD Stat. https://stats.oecd.org, retrieved on May 10, 2021.

Over ten years, China has been a key player in Africa in terms of lending. Thus, 47 out of 54 countries in Africa have received loans from China (https://chinaafricaloandata.bu.edu/). According to [36], over 150billion USD loan to Africa was committed by China between 2000 to 2018, and this amount is far above the amount committed by any other aid donors or loan within the period (see Fig 2 below). Following Belt and Road Initiative (BRI), some Africa countries have experienced increasing trade and foreign direct investments (FDI), and this increase in capital flows from China owing to BRI have been seriously criticized by many scholars, business analysts, US and wester media due the anxiety over Chinese lending prowess, and the belief that China is purposely indebting some Africa countries as a strategy to take control of key assets [36]. Hence, some analysts believed that this form of loans, often secured with valuable assets as collateral such as mineral resources and projects like port, is a deliberate attempt by the Chinese government to exploit the poorly indebted African countries–“dept-trap diplomacy”.

When these poorly and indebted countries defaulted and are unable to meet the debts obligations of the so called “predatory lending”, at times the assets could be seized thereby jeopardizing the economic progress of the country—example of such predatory lending is the instance of Hanbantota port in Sri Lanka in August 2017 and Ethiopia’s railway from Addis Ababa to Djibouti in 2018. Though, there are different conceptions or rather a lot of myths connected to Hanbantota port and Ethiopia’s railway contracts. The argument is that Sri Lanka government could not meet the external debt service obligations and following the concession agreement with CM Port, Hambantota Port was agreed to be managed as a Private Public Partnership (PPP) project with 70% shares to CM Port and 30% owned by the Sri Lanka Ports Authority (SLPA). Also, it was alleged that the business of the port is jointly handled by CM Port and SLPA, while the ultimate ownership structure remains the government of Sri Lanka. But the confusing and general myth is that Sri Lanka handed over the port project to China due to the inability to pay off the loan obtained, owing to the fact that the funds gained from the project were used to service Sri Lanka’s real balance of payment issues [37]. However [36], refuted the general belief that China-Sri Lanka port agreement is a debt-trap, claiming that the project is never a debt-equity swap, and was not at any time agreed to be pledged for the debt. Thus [36], perceived Sri Lanka port as a lease and posit that Sri Lanka government inability to pay off the debt is connected to its private bondholders. As such [38, 39], contradict [36] perceptions, believing that China initiates “debt trap” to swap cheap raw materials in poor economies to China, while other scholars such as [40–42] asserts that Chinese loans to Africa are oil-seeking strategies.

**Fig 3:**
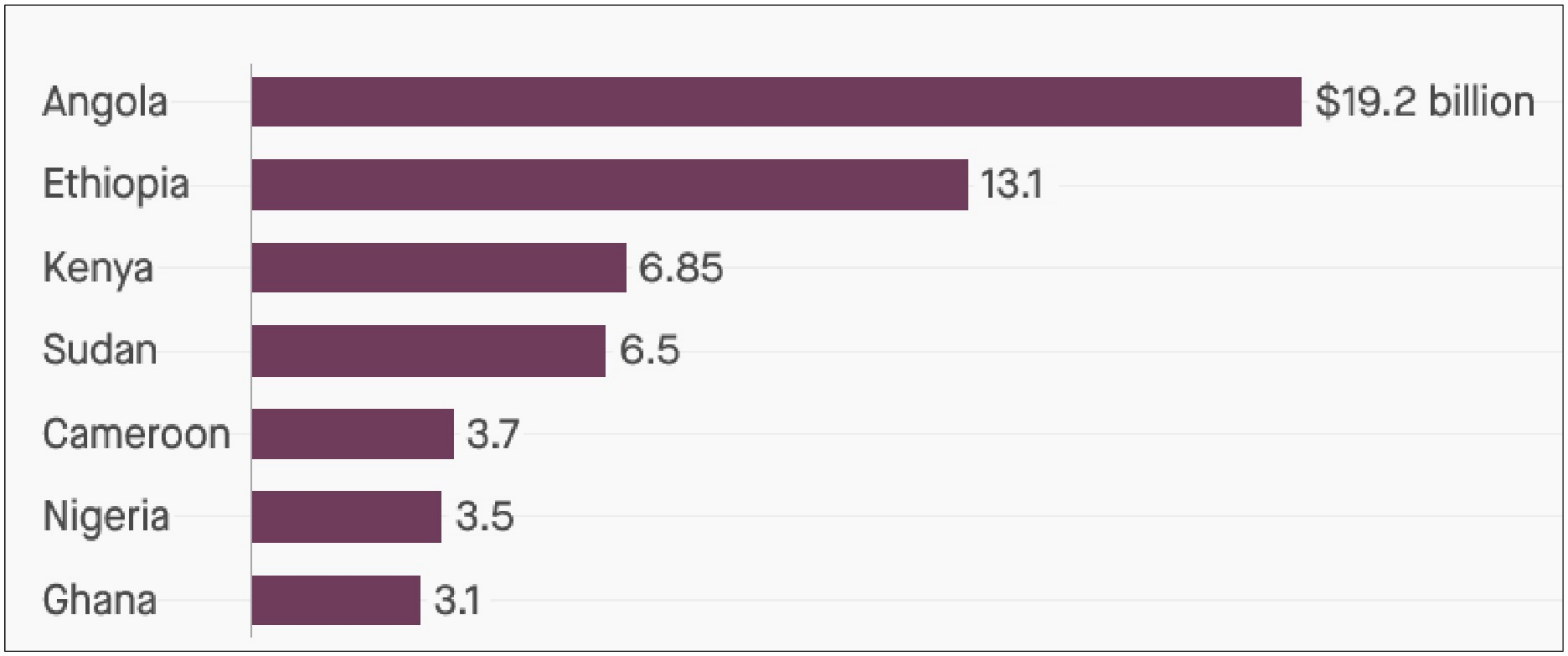
Chinese loans to Africa stylized fact (2000–2019). Sources: Plotted using Data from Johns Hopkins SAIS China-Africa Research Initiative.

Similarly, as the China’s second-largest African debtor (see Fig 3 below), Ethiopia benefited enormously from BRI partnership in infrastructure finance through the construction of first Ethiopia railway projects that linked Addis Ababa to Djibouti standard gauge railway (SGR). These projects were considered a case of “benefits and pitfalls” of Chinese finance [37]. Since the Ethiopia government was given the opportunity to deliberate on the terms of the loan by renegotiating the duration of the repayment of the loan which was originally fifteen (15) to thirty (30) years period [36], many scholars (e.g.,) perceived the idea as a great chance to benefits from the projects. However, the pitfall experience is associated to challenges of foreign exchange shortages, growing debt burden and deteriorating export performance which constrain the government of Ethiopia’s ability to repay numerous loans that financed these projects. Consequently, making the repayments on the principal for the Chinese railway loan that began in 2017 difficult and in early 2019, the Ethiopian Railway Corporation (ERC) was not only unable to redeem the loan from China but also not able to present the management fees balance to the Chinese companies operating the railway [36]. In view of the above arguments, there is no clear justification on the “myth of debt trap” or “dept-trap diplomacy” in Africa as noted by [43, 44] who refute the claim that Chinese loan is driven by natural resources. Also, China-Africa Research Initiative (CARI) condemned the idea that Chinese loans are targeted at resource-rich countries with the purpose of taking over the sovereign of the assets from the borrowing countries [36].

## 5. Review of related literature

Evidence has shown that a country can achieve economic growth in two ways. Growth may be an outcome of innovations resulting from competition or expansion of the level of investment according to [45] dynamic competition theory, and [46] neoclassical growth theory, respectively. The neoclassical growth theory argued that a country’s economic growth is dependent on her savings and investments. Hence [46], was of the view that policies that support greater savings could promote investment and economic growth. To support economic activities therefore, there must be sufficient funds, which apparently has been a challenge for most developing economies. Economic activities may be financed either internally/domestically (taxes), or externally (borrowing) if the internal sources of fund are not sufficient to finance budget deficits. On the other hand, the Dual Gap theory has been considered as having the best explanation as to why external financing is preferred to internal/domestic financing in achieving sustainable growth, especially when one considers the state of domestic savings in most developing countries. This theory was developed by [47], and it states that the growth of developing countries may be constrained by the existence of two gaps–the savings gap and the foreign exchange gap. A savings gap exists where there are low domestic savings which may result in savings falling short of the “required investment–which is investment necessary to grow at target rate”. To fill this gap therefore, the dual gap theory recommends that different policies which encourage inflow of foreign savings should be implemented. Such foreign savings may be in form of capital inflows that allow the developing country to invest more than it can save domestically. On the other hand, the theory cautions that owing to the low savings level and its consequences on aggregate investment, production and foreign earnings, there may be balance of payment deficit or import surpluses which in some cases constitute a foreign exchange gap. This gap may be filled with “aid flow” which could be in form of external borrowing as suggested by [46] neoclassical growth theory.

According to the neoclassical growth theory, external debt is positively related to economic growth. However, the theory stresses the need for the optimal allocation of the externally sourced fund in order to achieve the anticipated increase in investment that could transform any economy. In contrast, the debt overhang theory states that the positive and direct effects of external debt on economic growth may be negated at a point where additional debt could have a negative effect on economic growth. Thus, this theory supports the importance of external financing as a policy option to grow an economy, especially when the internal sources are insufficient as recommended by the neoclassical growth theory, but cautions that the marginal effect of the debt on economic growth be evaluated. The theory reflects the 1970s experience, when many Sub-Saharan Africa countries borrowed money with the intention of financing infrastructural and the industrial growth as part of an import substitution strategy, but were unsuccessful to optimally allocate the borrowed funds to promote investments which could generate employment and improve citizens’ welfare. Accordingly [4], argued that external debt does more harm than good [4]. Pointed out that debt has aided the financing of many unprofitable, unrealistic and low efficiency projects that have had negative impact on economic growth. Hence, while studies such as [13, 15, 16, 48–52] criticised the postulates of [46] and Dual Gap theory by [47], other studies such as [53–56] appear to concur with [46, 47] postulates.

[48] noted that external debt hinders economic growth due to the lack of information on the structure, nature and magnitude of the debt as well as difficulties in meeting debt obligations. In contrast [49], argued that high debt burdens result in unsustainable balance of payment deficits as well as huge budget deficits in most Sub-Saharan African countries [50]. In his empirical study on the impact of external debt on economic growth for 35 Sub-Saharan Africa, revealed that external debt has a negative impact on economic growth. This result supports the debt overhang hypothesis. Investigating 99 developing countries in Asia, Latin America, the Middle East and Sub-Saharan Africa [15], found external debt to be negatively related to economic growth which also confirms the debt overhang hypothesis. Studies such as [16, 51] also support these conclusions [16]. Provides evidence that a high debt stock reduces growth by suppressing capital accumulation and growth of total factor productivity. The study employed a panel data set of 93 developing countries over the period 1969 to 1998 [13]. Employed multivariate cointegration techniques to investigate the long and short run relationship between economic growth and external debt service in Turkey for the period 1956 to 1996, and discovered that a cointegrating relationship existed between external debt service and economic growth. In addition, external debt service was negatively related to economic growth. The study equally revealed that there was a uni-directional causality running from debt service to economic growth. Similarly [52], examined the dynamic relationship between external debt and economic growth in Pakistan between the years 1970 to 2003 while controlling for other sources of growth. The study employed a multiple cointegration procedure to examine the relationship between external debt and economic growth. The result revealed that in the long run, debt service affects gross domestic product negatively, mostly through its severe impact on capital and labour productivity. The result further showed a short and long run causality running from debt service to gross domestic product. Other studies such as [15, 30, 57, 58] were also against the postulates that external debt promotes economic growth.

[59] studied the external debt’s impact on economic growth, and also analysed the sustainability of the debt in Sub-Saharan Africa. Using a sample of 41 countries and annual time series data that covered the period 2000 to 2017, the estimated output from the panel threshold approach showed an inverse and significant relationship between external debt and economic growth. The study further investigated the sustainability of external debt using the bootstrapping method. The evidence revealed that external debt was unsustainable in SSA countries. Similarly [17], study found inconclusive evidence on the relationship between external debt and economic growth. Further examination showed an inverse relationship between external debt and economic growth in the lower threshold level of 30%. Investigating the existing relationship between corruption and external debt-growth [23], identified a positive and significant impact of corruption on external debt-growth. Extending the works of [17, 23, 24] examined corruption, shadow economy and government debt nexus for the period 1996 to 2012. The evidence suggests that the shadow economy is positively related to corruption in public debt, and reduces revenue from tax while encouraging increases in the debt burden. With the aid of GMM estimation techniques [60], studied the role of external debt and FDI in financial development in Africa. Using annual time series data that covered 2002 to 2015, their study revealed a significant positive relationship between external debt, FDI and financial development in Africa. However, they recommended that the authorities of the selected countries should put borrowed fund to more productive uses. Evidence from [25] study in selected MENA countries on weak institution and public debt revealed that institutional measures had significant impact on public debt accretion. They further observed that weak institutions were inversely related to fall in the growth rate of GDP [61]. Investigated the influence of institutional quality on public debt using GMM with a sample of 46 countries selected from in Sub-Saharan Africa over the period 2000 to 2014. The study found both direct and indirect relationships between public debt and institutional quality. Further examination showed a significant debt-growth relationship.

Other studies such as [5, 62, 63] investigated the impact of external debt and economic growth in Africa using different approaches. While [5] found an insignificant negative relationship between external debt and economic growth from a panel of 39 SSA countries over 1990 to 2013 using SGMM [62], study on six central Africa Economic and Monetary Community countries from 2000 to 2016 showed that public debt had a significant and indirect impact on economic growth. Using linear and non-linear panel ARDL approach [63], on the other hand, found a significant long-run relationship between external debt and economic growth when the non-linear ARDL approach was adopted, while the linear model revealed a significant long-run relationship between investment and economic growth. Also [18], examined the causal link between external debt and economic growth in selected ECOWAS member countries. Evidence from the study indicated the existence of short and long-run causal links between external debt and economic growth. Furthermore, investigating the relevance of external debt in investment and economic growth of 23 selected low-income countries for the period of 2000 to 2017 [19], found a significant inverse relationship between external debt, investment and economic growth using SUR model. In like manner [20], studied twelve selected countries from the emerging economies of Central and Eastern Europe for the period of 1995–2014 and found that external debt adversely affected economic growth in the long-run. Further investigation also showed a significant causal link between external debt and economic growth. [26] studied the institutional quality and external debt-growth nexus for the years 1996 to 2013 for 36 Sub-Saharan African countries using SGMM techniques. The study established the existence of a long-run relationship between external debt and economic growth. Further investigation also showed that institutional quality impacted on economic growth.

Similarly [64, 65], examined the motivation for post-reform evolution of the Chinese government. In this study, China’s Belt and Road Initiative (BRI) which focused on regional infrastructure projects in Eurasia and Africa (e.g., Kenya, Djibouti and Ethiopia) was examined. The findings of the study contradict the objective of BRI on regional infrastructural development in in the sampled countries. This study further argued that the purpose of this initiative may not be achieved due to the impending debt crisis and the outbreak of Covid-19. Thus, according to the study, the BRI projects may not be sustainable, and the expectations of economic transformation of Kenya, Djibouti and Ethiopia may be undermined. However, this study conclude that the impending debt crisis may disrupt the objective of such debt. Furthermore, due to the increasing controversy on China becoming Africa’s largest bilateral creditor [64]. Further investigates the consequence of debt-trap diplomacy and the after effect of excessive credit to debtor countries. Considering the effort of the International Monetary Fund (IMF) to resuscitate fiscal positions that are coherent with macroeconomic stability and maintain economic growth for the purpose of ensuring debtor compliance in some countries (e.g., Addis Ababa, Lusaka, and Nairobi) [64], argued that the occasional debt financing and distress are characteristic of unsuccessful assimilation of Africa into global market. Thus, in view of the assertion [64], call for further inquiry on the costs and benefits of current debt cycle in Africa with the emphasis on social, economic and politics behind the initiative. In a quest to ascertain the possibility of debt repayment, and inquire if China’s loan is a trap prioritizing the costs and benefits of the projects for China and Ethiopia [66], inferred that the growing indebtedness is capable of making Ethiopia economy to be more dependent on China, and thus concluded that China stands to gain more than Ethiopia. He further argued that the effect of debt burden could deter the repayment.

With the aid of macro econometric techniques [30], examined the impact of external debt on economic growth in Sub-Saharan Africa. The study revealed a significant and negative impact of debt overhang and crowding out effect on economic growth of the selected countries. The study concluded that excess external debts stock and external debt servicing affected investments in Sub-Saharan Africa. Similarly, in a cross-country study that comprised 99 developing countries from Sub-Saharan Africa, Latin America, Asia and Middle East [15], established a negative relationship between debt overhang and economic growth. According to their findings, direct channels such as current debt inflows as a ratio of GDP, past debt accumulation as a measure of debt overhang and debt service ratio were constraints to growth. Investigating the external debt-growth problem and capital flight [57], argued that poor growth is associated with high debt. However, the study linked this negative effect on high imposition of tax on capital which resulted in low rates of returns on capital, lower investment and growth. From their studies [58, 67], provided evidence of a negative relationship between debt service ratio and real GDP growth and investment respectively.

In addition [68], attempt to unravel the consequences of borrowing and the intending risk in Sub-Saharan Africa. They argued that in as much as global financial crisis and spending to compensate countercyclical policies have significantly reduce budget surplus across the region, increasing level of indebtedness has worsen the Sub-Saharan African economy which is inherent in high risk profile due to increasing public debt and debt service in the region. They further argued that the increasing risk profile may reduce the level for debt danger in the region. [69] undertake a multi-dimensional view on African futures, with particular reference to the presence and engagement of China in Africa economy using Rwanda as a case study. Though their study traced the origin of China-African partnership which started during the post-colonial context, but remain inconclusive on the implications of China’s involvement in the future of Rwanda’s economy. Because of the recent global trend [70], investigation on China’s motivations financing economies around the globe, particularly Africa. The study was motivated by the issue on debt sustainability. However, the evidence shows that uneven share of Chinese loan obligated to African countries with high credit risk levels, while more financial promises go to countries with lower levels of solvency in Africa unlike the Western donors. In like manner, adopting SGMM [71], linked debt and growth in fourty three African countries which span the period 2001–2018. Their emphasize was placed on analyzing the existing dynamic relationship. The evidence from the findings suggests the existence of long-run relationship between external debt and growth in Africa. This evidence also supported the recent argument by many scholars who perceived high rate of external debt as a contagion to growth in Africa [72, 73]. In addition, other studies on the effect of external debt on economic growth also revealed a negative and long-run effect of debt on growth [74, 75]. Their findings show that as external debt increases, economic growth get worse. Though, they further argued that domestic debt promotes economic growth.

Though many studies may have argued that external debt is negatively related to economic growth, but [53] had a different perspective and suggested that external debt can promote growth especially when borrowed funds are invested in sustainable projects that are capable of generating revenue for servicing the debt. Likewise [54], employed the panel group mean fully modified ordinary least square method to study the relationship between external debt and economic growth for six pacific island countries between the years 1988 to 2004. They found that a positive relationship existed between external debt and economic growth as such, a percentage increase in external debt stock led to a 0.25 percentage point increase in economic growth. In addition, the result utilised a vector error correction model and discovered that there was no granger causality between economic growth and external debt in the long run, but there was a significant causal relationship running from external debt to economic growth in the short run [55]. Employed two models to capture the linear and non-linear relationship between external debt stock and economic growth in Nigeria for the years 1975 to 2005. The Solow-type neoclassical growth model adopted in the study was modified following the work of [15]. For a more in-depth investigation [55], examined the impact of a large external debt stock with its servicing requirements and resulting fiscal deficit impact on private investment. Findings from the study showed that external debt contributes positively to economic growth up to a certain point after which the contribution becomes negative. Unfortunately, they failed to show the period within which the impact of the external debt impact became negative and their method for ascertaining this impact. However, the study inferred that savings compresses output due to the existence of debt overhang and crowding out effect. Though, the evidence on the outcome of the non-linear relationship between external debt and economic growth was not clear.

[76] decomposed external debt into public and private, and investigated the relationship between them, capital accumulation and production in selected Asian and Pacific countries. He carried out a granger causality test to confirm if his findings were consistent with the [77] and Dornbusch-Krugman propositions which state that, “external debt of developing countries is a symptom rather than a cause of economic slowdown” and “external debt causes economic slowdown” respectively. The outcome of the investigation led to the rejection of these hypotheses by Bulow and Rogoff, and Dornbusch-Krugman. However, the findings showed the overall effects of public and private debts on gross national product (GNP) to be small. Also, the feedback effect revealed an opposite sign indicating that growth in gross national product led to increase in public and private external debts. Interestingly, this finding supports the postulates of [46] neoclassical growth theory and other studies whose findings showed a positive relationship between external debt and economic growth, irrespective of the choice of economic growth measure. From the outcome of the study [76] argued that the positive and indirect effects of public external debt on gross national product showed that capital flight generated by rise in tax expectations was insignificant compared to the effect of public external debt investment financing on capital stock. Hence, he concluded that the effect of the public external debt on gross national product was positive and statistically significant.

[56] argued that shocks and management conflict, policy interactions and institutions (governance) play an important role in explaining debt accumulation and macroeconomic performance. From the in-depth review of literature, he observed that past studies emphasised more on the impact of external debt, external debts service and external debts shock on economic growth but forgot to examine the influence of institution/governance such as corruption control, rule of law, political instability, regulatory quality, government effectiveness and, voice and accountability among others on economic growth. These indicators are so important that they may distort or enhance the outcome of external debt of a country and its impact on economic interaction. Thus, we control for governance or institution’s influence on economic growth by establishing the interactive effects of institution and external debt on economic growth in selected Sub-Saharan African countries. The rationale for controlling for the influence of governance and examining the interactive effect of the measures in this study is based on the study of [28]. [22] pointed out that institutional quality/governance is an important factor that could explain the divergence in economic performances across developing economies. As such, countries with quality institution/governance where problems such as corruption and political instability are minimal can effectively utilise external debt compared to countries with poor levels of governance/institution. To explain the influence of quality institution/governance therefore, this research work controlled for the effect of governance/institution on external debt and economic growth, in order to establish their relationship in these countries. The study also investigated the interactive impact of external debt and measures of institutions/governance on the economic growth of these countries.

In a related study [78], inquired if public debt is useful or harmful on growth. The evidence from ARDL techniques of estimation show a long-run relationship between public debt and growth, suggesting that debt is more harmful to growth in South Africa. Investigating the relevance of institutional quality role in external-growth nexus in 53 selected countries [79], argued that the performance of debt on growth is highly dependent on the quality of institution. He further argued that the consequences of external debt on economic growth of any country is determined by the level of transparent of the institution, legal system, rule of law and government effectiveness in the enforcement of law. Hence, this evidence supports the findings of study by [21] on institutional quality and stock market development in Nigeria which also supported the role of viable institution in achieve growth and development. In another dimension, studies such as [80–83] among others also deliberated on African debt with emphasize on China-Africa relationship. This investigation was extended to the relationship between foreign aid and quality of governance; and the extent of its influence on per-capita income, revenue generation and economic growth.

[80] study reiterated on the increasing state of China-Africa relationship which is believed to leverage the economic, political and social status of the African continent. Though [80], dissertation findings contradict the motivation of the Africa engagement with the China. In like manner [81, 82], argued that the economic, political and social engagement with Africa is made possible because China wants to engage with Africa. In other words, if the partnership is not worth it, China may be uninterested in the engagement and thus, suggesting that China’s interest is always driven by their own domestic demands and economic benefits–for instance, China offers the largest amount of loans and bilateral to African countries. According to School of Advanced International Studies (Johns Hopkins) in collaboration with China-Africa Research Initiative (SAIS-CARI), between 2000 and 2018, Chinese investors committed about $153 billion to African public sector borrowers. Furthermore [84], criticized China’s idea as a “rogue donor” because of the provision of policy with no interest in understanding African problems. Thus [81], believed China’s intention to be more political oriented than economic oriented because of the divergent in China’s aid policy in Africa which depart largely from Western aid policies in terms of dollar value and other terms. Hence, Western aid policies are mostly concerned with improving the standards of governance and human rights at times, while China aid policy in Africa is characterised with trade agreements, which is basically on the acclaimed “infrastructural development” rather than interfering on the efficacy of the institutions in Africa to promote transparency that may consolidate the level of confidence of investors [81]. In support of [81, 84] view, Kagan (2006) pointed that China is consolidating severe, tyrannical and totalitarian rule in Africa thereby making a fuss of “informal league of dictators”, and thus worsening the African problem.

Contrary to the above assertion, irrespective of the intention of aid donor, the World Bank had argued that the problem of Africa is far beyond foreign aid, and reiterated that other than linking African economic situation to huge external debt—China’s aid, indicators such as governance and poor-quality institutions like feeble rule of law and government effectiveness, legal system, regulatory quality, corruption as well as voice and accountability should be addressed. This argument was based on the fact that, prior to the era of China-African partnership when aid levels in parts of Africa were very low during the past decade, most of the African countries were underperforming. Amidst [85], argued that one of the reasons governances in most of the African countries is poor arises from the fact that colonialism did little or nothing to develop a viable institution capable of addressing the demands of the contemporary states. In other to buttress the assertion [85], examined the relationship between foreign aid, institutions and governance in Sub-Saharan Africa controlling for tax, changes in per-capita income and political violence. The outcome of the findings shows that fall in economic growth and political violence impacted negatively on governance. Also, further evidence shows a negative relationship between aid and quality of government, suggesting that higher level of aid is inversely related to quality of governance as measured with the indexes of ICRG and tax revenues (% of GDP) in Africa. Furthermore [85], opined that the inability to control for political violence, could result to increase in the level of aid and this may worsen the governance or results to sharp fall tax revenues. Thus, warfare between opposing forces, especially a prolonged, bitter and sporadic struggle inherent in most of African countries attract caring aid and post conflict restoration assistance. In turn, this could result to weak institutions such as rules of law, bureaucratic quality, voice and accountability, regulatory quality, government effectiveness, legal system and corruption, and consequently result to significant reduction in economic growth (GDP). Therefore, increasing institutional quality and low aid volume with no effort to account for variation in per-capita income would likely exert inverse relation between dependence and the quality of governance [85]. Alternatively, deteriorating institutional quality and high aid volume with no effort to account for changes in per-capita income would also result to inverse relation between dependence and the quality of governance and in turn affect the revenue of African countries [85]. As such, aid play significant role in the development of a country if it is properly utilized and allocated in infrastructural development that can incentivize among others, inflow of foreign investment, job creation, increase in per-capita income and improved revenue generation.

[86] noted that African countries possess the economic potentials for revenue generation by broadening her tax system. They are of the view that shortfalls in African revenue that have partly caused high volume of external debt is not far from poor quality of governance and economic policies. On the other hand [83], insist that aid concentration in Africa may help transform her economy if a good and local model as well as viable economic environment is in place and thus, could help persuade the initiation of good economic policies and promote quality governance. Therefore, he believed that the reformation tax systems of African countries with viable macroeconomic environment would eventually increase the size of their tax receipts which can promote their revenue generation and economic growth. Hence, weak quality institutions encourage tax evasion and rent-seeking, while corruption adversely affect revenue [87–89].

## 6. Methodology

The study adopted the Dynamic System Generalised Method of Moment (SGMM) estimation technique, developed by [90, 91]. This estimation technique solves the problem of weak instruments associated with the difference Generalised Method of Moment (GMM) estimation technique. The SGMM is particularly reliable when the number of cross sections (N) is larger than the number of time series (T). It also solves the problem of endogeinity bias, reverse causality and the problems associated with omitted variables. According to [92], it controls for time effects *δ*_*t*_ as well as individual specific effects *π*_*i*_. According to [93], although GMM yields more efficient estimates compared to the two stage least square and the instrumental variable technique in the presence of heteroskedasticity; this study however, corrects for heteroskedasticity by applying the second step SGMM since the first step assumes homoscedasticity. We also employed the Hansen test for identifying restrictions in order to test for the validity of the instruments utilised in the model with 10 percent statistical significance level as our benchmark level of significance. Lastly, the models were estimated with robust standard errors which are consistent with panel specific autocorrelation.

Prior to estimation, the study employed descriptive statistics to summarise the mean, minimum, maximum and standard deviation of the variables in the model as well as the mean of the variables across the countries included in the model. This gives us an insight on the behaviour of the variables in each of the countries of interest. We also utilised a correlation analysis via the correlation matrix to estimate the degree of relationship amongst the variables in the model in order to avoid the problem of multicollinearity and derive unbiased coefficients.

### 6.1. Model specification and data

This study used annual time series data that spanned the period 1997 to 2020, covering thirty selected Sub-Saharan Africa countries. It is partinet to understand that the selected Sub-Saharan Africa countries were not based or grouped according to their historical income classification. Thus, our interest is basically on examining the impact of external debt on economic growth in Sub-Saharan Africa without preference to their historical income classification. Though, it is obvious that information on the magnitude of external debt impact on economic growth of low, middle and high income countries in the region would be of relevant to policy makers and government authorities. However, this limitations has been covered in our study which is ongoing. The variables of interest for the study were sourced from World Development Indicators [94] and World Governance Indicator [94] due to its availability. The econometric software for the estimation of the data set is Stata 15. Thus, in investigating the impact of external debt on economic growth in Sub–Saharan Africa, the study specifies a dynamic model where;

$${GDP}_{it}=\alpha_{0}+\alpha_{1}{GDP}_{t-1}+\alpha_{2}{DEBT}_{it}+\alpha_{3}X_{it}+\delta_{t}+\pi_{i}+\epsilon_{it}$$ (1)

where *GDP*; Gross Domestic Product in constant US$, *GDP*_*t*–1_; the first-year lag of GDP, *DEBT*; external debt proxied with external debt stocks measured as % of Gross National Income (GNI). In addition, *X* is a set of control variables which includes capital and labour. Their inclusion in the model is based on the neoclassical production function which states that both variables are the main determinant of output (GDP) in an economy. We proxy capital with the gross fixed capital formation in constant US$ and labour with population, ages 15–64 as a percentage of total population. Eq (1) can thus be re-expressed as;

$${GDP}_{it}=\alpha_{0}+\alpha_{1}{GDP}_{t-1}+\alpha_{2}{DEBT}_{it}+\alpha_{3}{Capital}_{it}+\alpha_{4}{Labour}_{it}+\delta_{t}+\pi_{i}+\epsilon_{it}$$ (2)

To understand the influence of governance on external debt and in the economic growth relationship, we introduce [27] governance indicators namely, corruption control, government effectiveness, political stability, regulatory quality and, voice and accountability. These indicators were interacted with external debt to examine their influences on economic growth in order to determine the relevance of viable legal systems or institutions, and the political environment in Sub-Saharan Africa. Hence, we modify Eq (2) as;

$${GDP}_{it}=\alpha_{0}+\alpha_{1}{GDP}_{t-1}+\alpha_{2}{DEBT}_{it}+\alpha_{3}{Capital}_{it}+\alpha_{4}{Labour}_{it}+\alpha_{4}{interact}_{it}+\delta_{t}+\pi_{i}+\epsilon_{it}$$ (3)

$${GDP}_{it}=\alpha_{0}+\alpha_{1}{GDP}_{t-1}+\alpha_{2}{DEBTVOL}_{it}+\alpha_{3}{Capital}_{it}+\alpha_{4}{Labour}_{it}+\alpha_{4}{interact}_{it}+\delta_{t}+\pi_{i}+\epsilon_{it}$$ (4)

In Eq (4), *DEBTVOL* represents external debt volatility which we capture with the standard deviation of external debt as % of GNI. Hence, *δ*_*t*_ is the time specific effect, while *π*_*i*_ is the country specific effect. *ϵ*; represents the error term while *i* indicates the cross sectional index. Thus, *t* is the time index. For ease of interpretation, we transform economic growth and capital to their natural logarithm. The study covers thirty selected Sub-Saharan African countries for the years 1997 to 2020 based on available data. The countries selected for the study include: Benin, Botswana, Burkina Faso, Burundi, Cape Verde, Cameroon, Congo DR, Cote d’Ivoire, Ethiopia, Gambia, Ghana, Guinea-Bissau, Kenya, Lesotho, Liberia, Madagascar, Mali, Mauritania, Mauritius, Malawi, Niger, Nigeria, Senegal, Sierra Leone, Rwanda, South Africa, Swaziland, Tanzania, Togo, Uganda.

## 7. Presentation and discussion of results

We begin this section by describing the data in the model. Results in Table 3 show that economic growth in sub–Saharan Africa has a mean value of 9.918, with a standard deviation of 0.963. Economic growth also has a minimum value of 0.001 and a maximum value of 11.87. Also, debt has a minimum value of 0.011 and a maximum value of 610.45. This shows that economic growth and debt in its natural logarithm is quite dissimilar across Sub–Saharan African countries, and suggests that countries in Sub–Saharan Africa are growing at a different rate. Table 4 supports this conclusion as can be seen from their mean values which are different for all countries under empirical observation. Table 4 also shows that Swaziland has the highest level of economic growth in sub–Saharan Africa with mean value of about 11.69 followed closely by South Africa (11.40), Nigeria (11.34) and Kenya (10.51), while Liberia, Guinea-Bissau and Gambia had the lowest level of economic growth with mean value of 8.102, 8.84 and 9.06 respectively. Similarly, capital in Table 3 has a mean value of 20.954, a minimum value of -2.424 and a maximum value of 53.99. Both the minimum and the maximum values are somewhat far away from the mean value which denotes dissimilarity in the level of capital in their natural logarithm in sub–Saharan Africa, as is the case of economic growth. While Mozambique, Botswana and Mauritania had the highest level of capital with mean value of 32.25, 32.46 and 31.39 respectively, Guinea-Bissau, Burundi and Gambia had the lowest level of capital with mean value; 11.69, 11.73 and 13.26 respectively as shown in Table 4.

**Table 3:** Descriptive statistics of the variables in the model.

Variables	Acronym	Obs	Minimum	Maximum	Mean	Standard Deviation
Economic Growth	E.Growth	720	0.001	11.87	9.918	0.963
Capital	Capital	720	-2.424	53.99	20.954	9.751
Labour	Labour	720	5.128	7.801	6.594	0.573
Debt	Debt	720	0.011	610.5	56.92	65.66
Control of corruption	CC	720	-1.700	2.100	-0.406	0.928
Government effectiveness	GE	720	-2.200	1.036	-0.493	0.973
Political stability	PS	720	-2.665	1.598	-0.309	0.913
Regulatory quality	RQ	720	-2.450	7.001	-0.086	1.379
Voice/accountability	VA	720	-2.280	4.021	-0.538	1.023

Source: Authors’ computation: Economic Growth, Capital, Labour and Debt are in log form.

**Table 4:** Mean value of variables across countries (1997–2020).

S/N	Country	Growth	Capital	Labour	Debt	Gov	S/N	Country	Growth	Capital	Labour	Debt	Gov
1	Benin	9.88	17.99	6.55	25.00	-0.45	16	Mauritius	9.914	22.78	5.747	81.51	-0.12
2	Botswana	10.02	31.46	5.89	10.38	-0.37	17	Mauritania	9.607	31.39	5.965	59.32	-0.03
3	Burkina Faso	9.88	18.91	6.76	28.67	-0.46	18	Mali	9.903	20.38	6.723	47.33	-1.04
4	Burundi	9.21	11.73	6.56	73.77	-0.47	19	Malawi	9.665	29.76	6.769	59.32	-1.26
5	Cape Verde	9.08	23.85	5.25	62.78	-0.94	20	Mozambique	9.999	32.25	7.008	88.83	-1.04
6	Cameroon	10.33	22.47	6.94	44.88	-0.84	21	Niger	9.77	21.72	6.784	34.52	-0.17
7	Congo DR	10.27	15.25	7.35	78.60	-0.70	22	Nigeria	11.34	24.34	7.696	20.98	-0.92
8	Cote d’Ivoire	10.38	13.75	6.83	66.20	0.43	23	Rwanda	9.656	18.78	6.675	43.74	-0.45
9	Gambia	9.06	13.26	5.74	53.53	1.09	24	Senegal	10.12	24.59	6.526	39.03	0.82
10	Ghana	10.34	21.78	7.00	56.86	-0.96	25	Sierra Leone	9.328	13.37	6.323	78.68	-0.15
11	Guinea-Bissau	8.84	11.69	5.78	154.5	-1.06	26	South Africa	11.40	18.66	7.280	29.64	-0.81
12	Kenya	10.51	18.50	7.21	33.24	0.668	27	Swaziland	11.69	25.88	6.646	41.13	-0.39
13	Lesotho	9.214	15.67	5.957	37.42	-1.04	28	Tanzania	10.42	29.58	7.308	38.64	-0.66
14	Liberia	8.102	17.33	6.196	174.9	-0.19	29	Togo	9.493	20.22	6.315	56.64	-0.24
15	Madagascar	9.919	18.44	6.996	48.96	-0.53	30	Uganda	10.19	23.03	7.049	37.02	-0.39

Source: Authors’ computation.

Note: Mean values were estimated for the years 1997–2020 for each country. Growth is Economic Growth while Gov is Governance. Governance was computed as the average of the five indicators employed in the study for conciseness, and countries with positive values seems to have viable government or better institutions.

Also, the labour force in Nigeria, Democratic Republic of Congo and Tanzania are higher than in other countries in sub–Saharan Africa with a mean value of 7.69, 7.35 and 7.31 respectively, while it was lowest in Cape Verde, Gambia and Mauritius with a mean value of 5.25, 5.74 and 5.75 respectively. For the total sample as indicated in Table 3, the mean value of labour is 6.594. Result for debt indicates that while its mean value is 56.92, it’s minimum and maximum are 0.011 and 610.45 respectively. This large disparity between the minimum and the maximum value represents the large difference in the amount of external borrowing across the sample of countries in the model. While Liberia, Guinea-Bissau and Mozambique had the highest mean value of external debt as indicated in Table 4 with a value of 174.9, 154.5 and 88.83 respectively, Botswana, Nigeria and Benin republic had the lowest mean value of the external debt with values of 10.38, 20.98 and 25.00 respectively.

Table 4 also presents the mean value of governance for the countries in the sample which was computed as the average of the five indicators employed in the study. Results show that countries like Cote d’lvoire, Gambia, Kenya, and Senegal relatively have better institutions compared to other countries as indicated by their positive values, while countries like Cameroon, Congo DR, Cote d’Ivoire, Guinea-Bissau, Lesotho, Liberia and Nigeria among others have the weakest governance quality in sub–Saharan Africa. For the total sample of countries in Table 4, the result shows that generally, sub–Saharan Africa has a weak level of governance or institutional quality as indicated by the negative mean values of the indicators of governance.

A review of the correlation matrix presented in Table 5 discloses the mutual or complimentary relationship between the variables in the model. From the results, we observed that capital and labour have a positive correlation with economic growth, while external debt (Debt) has a negative correlation with economic growth. Also, all the indicators of quality governance or institution have negative correlation with economic growth except for government effectiveness (GE) which exhibit positive correlation with economic growth. Further investigation also revealed that indicators such as government effectiveness and, voice and accountability (VA) strongly and positively correlated with corruption control (CC), while VA exhibit positive and strong correlation with GE. Similarly, these governance indicators also have negative correlation with capital and labour. Hence, similar trend is observed in the correlation between governance indicators and external debt except for the political stability (PS). This simply show that weak governance or institution may likely deter economic growth, capital, labour and could worsen external debt in Sub-Saharan Africa. The indicators of governance in this study do not enter the model directly but through its interaction with external debt. This is to enable us evaluate the interactive influence of governance and external debt on economic growth in Sub-Saharan Africa.

**Table 5:** Correlation matrix.

Variable	E.Growth	Capital	Labour	Debt	CC	GE	PS	RQ	VA
E.Growth	1.0000								
Capital	0.297	1.0000							
Labour	0.557	0.099	1.0000						
Debt	-0.272	-0.159	-0.230	1.0000					
CC	-0.034	-0.044	-0.039	-0.041	1.0000				
GE	0.079	-0.088	-0.075	-0.016	0.816	1.0000			
PS	-0.036	-0.057	-0.135	0.018	0.519	0.463	1.0000		
RQ	-0.029	-0.080	-0.056	-0.009	0.485	0.4563	0.311	1.0000	
VA	-0.032	-0.102	-0.037	-0.014	0.829	0.8026	0.519	0.439	1.0000

Source: Authors’ computation. Note: CC is control of corruption; GE is Government Effectiveness; PS is Political Stability; RQ is Regulatory Quality; VA is Voice/Accountability.

Table 6 presents the results of the estimated models using the dynamic system generalized method of moments (SGMM). The results reveal that the one-year lag of economic growth has a positive and significant impact on contemporaneous economic growth at 1 percent level of significance which is true across all columns of the results. This is logical as previous levels of economic growth can increase current economic growth through channels such as increase in available capital for savings and investment purposes as well as the increase in aggregate demand. In addition, the result reveals that capital has a positive and significant impact on economic growth in Sub–Saharan Africa across the models. However, a percentage increase in capital leads to a more than 0.6641percentage point increase in economic growth. This finding supports the postulates of [46] which emphasises the need for greater savings (source of capital) and its implication for investment and economic growth. The same point was equally reiterated by Keynes who viewed investment as a function of savings. Further inspection shows that labour is positively and significantly related to economic growth in sub–Saharan Africa across the models. This implies that a percentage increase in labour leads to a more than 0.27249 percentage point increase in economic growth. This outcome signifies that labour is an important determinant of economic growth. Hence, the quality and the rate of growth of labour is vital for economic progress, and the role could be extended through severe investment in human capital development or training. Amidst, making the labour force an evolving talent pool would make the production process more efficient and in turn, stimulate the rate of growth. This result support a priori expectations, and [46] who believed in policies that could support greater investment and economic growth. Therefore, the overwhelming population of the region could be an advantage because of the availability of cheap labour and the fact that most of the Sub-Saharan African countries are labour intensive.

**Table 6:** External debt and economic growth.

Variables	(1)	(2)	(3)	(4)	(5)	(6)
Constant	0.259* (0.126)	0.168*** (0.008)	0.082** (0.020)	0.123*** (0.007)	0.499** (0.013)	0.768** (0.023)
E.Growth (-1)	0.664*** (0.000)	0.629*** (0.000)	0.600*** (0.000)	0.674*** (0.000)	0.670*** (0.000)	0.626*** (0.000)
Capital	0.218** (0.040)	0.232** (0.032)	0.242** (0.018)	0.196** (0.048)	0.217* (0.065)	0.238*** (0.010)
Labour	0.273*** (0.005)	0.270*** (0.000)	0.306*** (0.002)	0.266*** (0.004)	0.274*** (0.002)	0.278** (0.018)
Debt	- 0.053* (0.089)					
Debt*CC		0.014*** (0.002)				
Debt*GE			0.0014** (0.030)			
Debt*PS				0.0301*** (0.006)		
Debt*RQ					0.00013*** (0.004)	
Debt*VA						0.00092** (0.016)
Obs	690	690	690	690	690	690
AR(1)	(0.035)**	(0.022)**	(0.020)**	(0.027)**	(0.0251)***	(0.027)**
AR(2)	(0.615)	(0.432)	(0.616)	(0.471)	(0.602)	(0.712)
Hansen	(0.210)	(0.311)	(0.300)	(0.511)	(0.371)	(0.411)
Wald	(0.000)*	(0.000)*	(0.000)*	(0.000)*	(0.000)*	(0.000)*

Source: Authors’ computation. Economic growth is the dependent variable.

***, ** and * denotes statistical significance at 1%, 5% and 10% respectively. E.Growth denotes economic growth. (.) is the probability values, and the models were estimated with robust standard error.

The results on the impact of external debt on economic growth in sub–Saharan Africa as shown in the first column of Table 6 also revealed that external debt had a negative and significant impact on economic growth (E.Growth) in sub–Saharan Africa. This implies that a percentage increase in external debt leads to a—0.05281 decline in economic growth at ten percent significant level. This suggests that external debt on its own may retard economic growth in sub–Saharan Africa. This finding contradicts the postulates of the neoclassical growth theory and overhang theory which argued that external debt promotes economic growth. Our finding is consistent with studies by [4, 15, 16, 48–50]. However, the empirical evidence from [53–56] agree with [46] and overhang theory postulates. Considering the relevance and role of governance in every economy, we further investigate the interactive influence of governance and external debt on economic growth. The interactive results of external debt (Debt) and governance indicators are presented in column 2–6 in Table 6. There was no problem of serial correlation as the results across columns 1–6 show that AR(2) is satisfactory indicating that the models are free from serial correlation. To verify the validity of the instrument, we used the Hansen test which confirmed that the instruments employed in the study are valid. The Wald test result also reveals that the explanatory variables in the model have joint and significant impact on economic growth in sub–Saharan Africa at one percent level of significance.

From Table 6 also, we observed that the interaction of external debt (Debt) with corruption control (CC) leads to a positive and significant impact on economic growth (E.Growth) in Sub-Saharan Africa. This suggests that the impact of external debt on economic growth would be positive when corruption is adequately controlled. This result shows the extent to which an improvement in corruption control can influence the external debt and economic growth relationship in the region. Also, when government effectiveness, as shown in column 3, interacts with external debt, it produces a positive and significant influence on economic growth. Though, the magnitude of impact of the interactive variables, Debt*CC and Debt*GE as explained by their coefficients values, 0.0144 and 0.00143 respectively is not as severe as the direct impact of external debt on economic growth. Hence, the interactive influence supports the need for an improved governance in the region. The result further reveals that an improvement in political stability (column 4) and regulatory quality (column 5) results in a positive and significant relationship between external debt and economic growth. Finally, the interaction between voice and accountability and debt in column 6 significantly influence economic growth. These results imply that the quality of governance or institution is a strong factor that could magnify the benefits of external debt in promoting economic growth in sub–Saharan Africa.

These results may explain why many bilateral, multilateral and other form of lending into Africa have not had significant improvement in infrastructural development in the region. According to ICA reports, in 2018, a total sum of $100.8bn was committed to finance infrastructural development in Africa, which is about 24% increase of the total amount received in 2017 ($81.6bn) and 38% increase on the $75.8bn received three previous years from 2017. Hence, out of $100.8bn received in 2018, about $37.5bn (37%) share were committed by African governments, $25.7bn (26%) from China and $20.2bn (20%) from ICA members. Amid the huge financing over the years, infrastructure investment still remains a serious challenge to economic growth in the region and this has affected industrial growth despite large borrowings. This underscores the importance of quality governance or viable institutions in the region. Thus, according to [21, 22, 28], quality governance or viable institutions could enhance the efficient use of external debt and promotes growth through the creation of an enabling environment. The study also tested for the impact of external debt volatility on economic growth. In this study, external debt volatility was captured by its standard deviation. In like manner, we investigated the interactive influence of governance or institutional indicators and external debt volatility on economic growth in sub–Saharan Africa. From the results presented in Table 7, we observed that external debt volatility had a negative and significant impact on economic growth in the region. Hence, its impact is not as severe as the direct effect of external debt on economic growth as indicated by its coefficient.

**Table 7:** External debt volatility and economic growth.

Variables	(1)	(2)	(3)	(4)	(5)	(6)
Constant	0.688*** (0.011)	0.496*** (0.002)	0.527*** (0.002)	0.479*** (0.001)	0.746*** (0.010)	0.483*** (0.015)
E.Growth(-1)	0.680*** (0.000)	0.674*** (0.000)	0.676*** (0.000)	0.690*** (0.000)	0.671*** (0.000)	0.679*** (0.000)
Capital	0.184* (0.063)	0.199* (0.091)	0.199* (0.080)	0.193* (0.075	0.2082* (0.075)	0.218* (0.080)
Labour	0.258*** (0.001)	0.252 (0.006)	0.252*** (0.002)	0.254*** (0.001)	0.267*** (0.003)	0.267*** (0.001)
Debtvol	-0.0025* (0.058)					
Debtvol*CC		0.0024*** (0.001)				
Debtvol*GE			0.0023*** (0.002)			
Debtvol*PS				0.0389*** (0.008)		
Debtvol*RQ					0.0009*** (0.008)	
Debtvol*VA						0.0684*** (0.005)
AR(1)	(0.030)**	(0.071)*	(0.041)**	(0.052)*	(0.032)**	(0.020)**
AR(2)	(0.720)	(0.930)	(0.960)	(0.311)	(0.760)	(0.791)
Hansen	(0.341)	(0.292)	(0.591)	(0.238)	(0.541)	(0.210)
Wald	(0.000)*	(0.000)*	(0.000)*	(0.000)*	(0.000)*	(0.000)*

Source: Authors’ computation. Economic growth is the dependent variable.

***, ** and * denotes statistical significance at 1%, 5% and 10% respectively. E.Growth denotes economic growth. (.) is the probability values, and the models were estimated with robust standard error. Debtvol denotes external debt volatility.

In addition, the results of the interactive effects of external debt volatility and governance or institution on economic growth also show that all the indicators of governance have a positive and significant influence on economic growth in sub–Saharan Africa. However, while voice and accountability (VA = 0.06838), and political stability (PS = 0.03896) exhibits significant and largest influence on the relationship between external debt volatility and economic growth, regulatory quality (RQ = 0.00088) had the lowest influence. In addition, government effectiveness (GE = 0. 00234) and corruption control (CC = 0.00242) are observed to significantly exert positive influence on the relationship between external debt volatility and economic growth. Hence, while the interactive influence of Debtvol*VA and Debtvol*PS were found to be more severe on economic growth in the region in terms of their coefficients, the interactive influence of Debtvol*CC and Debtvol*GE on economic growth remains significant and positive. This implies that quality governance accompanied with corruption control, government effectiveness, voice and accountability, political stability and regulatory quality promotes viable regulatory environment that is capable of encouraging transparency, enforcement of law and order, and checks and balances. Hence, this may help in managing debt crisis that usually occur when debt obligations are defaulted, and thus reduce the shocks on the economy. The results also revealed that capital and labour positively influenced economic growth. Overall, there is no problem of serial correlation and the Hansen test p-value reveals that the instruments are valid with the Wald test showing joint significance of the regressors across all columns.

## 8. Conclusion, policy implications and recommendations

External debt is perceived to be an important revenue source by many developing countries to augment domestic sources for economic growth and development. However, most Sub-Saharan African countries have failed to effectively utilise these external funds to improve their economies, with some even becoming worse-off, and experiencing challenges in form of debt overhang and liquidity constraints, amongst other. This study examined the impact of external debt on economic growth in thirty selected Sub-Saharan African countries for the period 1997 to 2020. Furthermore, the study accounted for the effect of external debt shocks by investigating the impact of external debt volatility on economic growth in the region. Thus, the findings show that external debt and external debt volatility had negative impacts on economic growth, implying that huge borrowings and future debts, dereliction and fungibility of funds as well as non-use of borrowings for capacity development deters economic growth in the region. The study however, revealed that notwithstanding its volatility, external debt can in fact have a positive impact on economic growth in Sub-Saharan Africa when there is an improvement in the quality of governance or institution as shown in Tables 6 and 7 above. In addition, the findings from the interactive effect of governance and external debt on economic growth, accounted for the influence of quality government which studies such as [5, 17, 19], do not consider necessary. Hence, the evidence show that quality governance influences external debt and impact positively on economic growth, suggesting that quality governance matter in promoting efficient use of external debt in the region. As such, further inspection on the interactive effect of external debt volatility and governance on economic growth also supported our earlier findings on the interactive effect of governance found missing in [25, 26] which focussed on MENA countries. Amidst, external debt and external debt volatility impacted positively and significantly to economic growth when interacted with the measures of governance, indicating that quality government matter in promoting efficient allocation resources.

The implication of these findings is that there should be a limit to borrowings as acknowledged by [95], who argued that the Guidotti-Greenspan rule is a significant determinant of reserve holdings. This would allow reserves to create a benchmark for borrowings and other debt accruals. Again, the findings suggest that economies with vulnerability in their capital accounts could experience crisis especially if external funds are not properly utilized. Thus, a sound analysis of the economic and social profitability of all debt-financed projects is important before to ensure that the returns generated will be in excess of the interest and capital repayments before acquiring such loans [31]. African countries should ensure that the quality of governance can support the effective and efficient allocation of all external debts for the purpose of promoting growth in their various countries. Furthermore, the five indicators of governance employed in the study and their interaction with external debt, as well as external debt volatility shows that there is a positive and significant relationship between external debt, its volatility and economic growth in the presence of improved governance. The study therefore recommends that when using borrowed funds, African leaders should pay more attention to governance quality as this can curb most of the ills of the region as indicated in the estimated results.

Since the interactive effect of governance indicators with external debt was found to be positive and significant, it shows that an efficient and effective government is vital for economic growth. This could be achieved by strengthening institutions. It is essential for Sub-Saharan African countries to introduce policies that will improve the quality of their legal systems, as well as the public and civil services. The independence of the civil and public service should be encouraged and political interferences kept to a minimum to enable sound policy formulation and implementation. Most importantly, Sub-Saharan African governments should be committed to policies that can reduce the widespread corruption devastating many African economies. Accordingly, improved control of corruption, political stability, viable legal systems, freedom of expression and association, as well as an environment where people participate in selecting their government will enhance economic growth. Sub-Saharan African governments should direct these external funds to projects that can create new opportunities for investment and attract more investors into the region.

## Supporting information

S1 Appendix (DOCX)
