# Supplementary material for: External debt and economic growth in Sub-Saharan Africa: Does governance matter?
Source: PLoS One. 2022 Mar 4;17(3):e0264082. doi: 10.1371/journal.pone.0264082 (PMC9067604; doi:10.1371/journal.pone.0264082)
Supplement: S1 Appendix — (DOCX) [file pone.0264082.s001.docx]

**Appendix**

**Table 8: Definition of variables.**

| **Variable** | **Functional Definition** | **Data Source** |
| --- | --- | --- |
| Economic Growth | This is an increase in the capacity of an economy to produce goods and services, compared from one time period to another. | WDI (2020) |
| External Debt | This refers to money borrowed from a source outside the home country. | WDI(2020) |
| Capital (Gross Capital Formation) | Capital refers to funds which are available for investment and production purposes. | WDI(2020) |
| Labour | Labour is the total of all human physical and mental effort used in creation of goods and services. | WDI(2020) |
| Control of corruption | Control of corruption is the perceptions of the extent to which public power is exercised for private gain, including both petty and grand forms of corruption, as well as the of the state by elites and private interests. Estimate gives the country's score on the aggregate indicator, in units of a standard normal distribution, i.e. ranging from approximately -2.5 to 2.5. | WGI(2020) |
| Government effectiveness | Government Effectiveness captures perceptions of the quality of public services, the quality of the civil service and the degree of its independence from political pressures, the quality of policy formulation and implementation, and the credibility of the government's commitment to such policies. Estimate gives the country's score on the aggregate indicator, in units of a standard normal distribution, i.e. ranging from approximately -2.5 to 2.5. | WGI(2020) |
| Political Stability | Political Stability and Absence of Violence/Terrorism measures perceptions of the likelihood of political instability and/or politically-motivated violence, including terrorism. Estimate gives the country's score on the aggregate indicator, in units of a standard normal distribution, i.e. ranging from approximately -2.5 to 2.5. | WGI(2020) |
| Regulatory Quality | Regulatory Quality captures perceptions of the ability of the government to formulate and implement sound policies and regulations that permit and promote private sector development. Estimate gives the country's score on the aggregate indicator, in units of a standard normal distribution, i.e. ranging from approximately -2.5 to 2.5. | WGI(2020) |
| Voice/Accountability | Voice and Accountability captures perceptions of the extent to which a country's citizens are able to participate in selecting their government, as well as freedom of expression, freedom of association, and a free media. Estimate gives the country's score on the aggregate indicator, in units of a standard normal distribution, i.e. ranging from approximately -2.5 to 2.5. | WGI(2020) |
